# Vaccination with DNA Encoding Truncated Enterohemorrhagic *Escherichia coli* (EHEC) Factor for Adherence-1 Gene (efa-1′) Confers Protective Immunity to Mice Infected with *E. coli* O157:H7

**DOI:** 10.3389/fcimb.2015.00104

**Published:** 2016-01-20

**Authors:** Roberto Riquelme-Neira, Alejandra Rivera, Darwin Sáez, Pablo Fernández, Gonzalo Osorio, Felipe del Canto, Juan C. Salazar, Roberto M. Vidal, Angel Oñate

**Affiliations:** ^1^Laboratory of Molecular Immunology, Department of Microbiology, Faculty of Biological Sciences, Universidad de ConcepciónConcepción, Chile; ^2^Microbiology and Mycology Program, Faculty of Medicine, Institute of Biomedical Sciences, University of ChileSantiago, Chile

**Keywords:** DNA vaccine, truncated efa-1 gene, enterohemorragic *E. coli*, O157:H7 serotype, protective to mice

## Abstract

Enterohemorrhagic *Escherichia coli* (EHEC) O157:H7 is the predominant causative agent of hemorrhagic colitis in humans and is the cause of haemolytic uraemic syndrome and other illnesses. Cattle have been implicated as the main reservoir of this organism. Here, we evaluated the immunogenicity and protective efficacy of a DNA vaccine encoding conserved sequences of truncated EHEC factor for adherence-1 (*efa-1*′) in a mouse model. Intranasal administration of plasmid DNA carrying the efa-1′ gene (pVAX*efa-1*′) into C57BL/6 mice elicited both humoral and cellular immune responses. In animals immunized with pVAX*efa-1*′, EHEC-secreted protein-specific IgM and IgG antibodies were detected in sera at day 45. Anti-EHEC-secreted protein sIgA was also detected in nasal and bronchoalveolar lavages. In addition, antigen-specific T-cell-proliferation, IL-10, and IFN-γ were observed upon re-stimulation with either heat-killed bacteria or EHEC-secreted proteins. Vaccinated animals were also protected against challenge with *E. coli* O157:H7 strain EDL933. These results suggest that DNA vaccine encoding *efa-1*′ have therapeutic potential in interventions against EHEC infections. This approach could lead to a new strategy in the production of vaccines that prevent infections in cattle.

## Introduction

Most strains of *Escherichia coli* form part of the normal microbiota of the gastrointestinal tract of mammals and birds. However, several highly adapted *E. coli* strains possess special virulence properties, allowing them to adapt to alternative niches and to cause a broad spectrum of diseases including diarrhea. These strains, named diarrheagenic *E. coli* (DEC), have been classified based on their known virulence properties (Kaper et al., 2004). Enterohemorrhagic *E. coli* (EHEC) are important intestinal zoonotic pathogens, causing sporadic, and epidemic *E. coli* infection outbreaks worldwide (Bosilevac and Koohmaraie, 2011). EHEC is a type of Shiga toxin-producing *E. coli* (STEC) that colonizes the human intestine and causes diarrheal illness that can progress to hemorrhagic colitis and, in several cases, life-threatening haemolytic uremic syndrome (HUS) (Kaper et al., 2004; Farfan and Torres, 2012). Ruminants, especially cattle, are the primary source of STEC O157:H7, the most commonly detected strain, and harbor non-O157 STEC strain (Pennington, 2010; Bosilevac and Koohmaraie, 2011), and beef is considered to be an important source of STEC O157 and non-O157 human infection (Caprioli et al., 2005).

EHEC and enteropathogenic *E. coli* (EPEC) are intestinal pathogens that have the ability to form attaching and effacing (A/E) lesions in host intestinal epithelium (Schmidt, 2010). A/E lesions are characterized by bacterial attachment with the formation of an actin pedestal-like structure and by destruction of epithelial microvilli (Goosney et al., 2000). This pathology is genetically determined by the locus of enterocyte effacement (LEE) (Crawford et al., 2002; Kaper et al., 2004), which is highly conserved in EHEC and EPEC. The LEE contains a significant number of genes associated with virulence, mainly encoding a type III secretion system (T3SS), and the eae gene encoding the outer membrane adhesin intimin that, along with the translocated intimin receptor (Tir), allows intimate bacterial binding to intestinal epithelium (Crawford et al., 2002). However, some genes coding for effector proteins and associated factors implicated in EPEC and EHEC pathogenesis are located outside the LEE island, forming part of a large pathogenicity island responsible for increased virulence (Klapproth and Meyer, 2009). In various EHEC and EPEC strains, these include lifA/efa-1 (lymphocyte inhibitory factor A/EHEC factor for adherence-1), that encodes a toxin of approximately 360 kDa, one of the largest proteins produced by *E. coli.* It contains glycosyltransferase and protease domain, both present also in Clostridial cytotixins (Klapproth, 2010). LifA/Efa1 protein has been detected at the surface of the EPEC JPN15 strain (Badea et al., 2003) and affects intestinal colonization and adhesion by modulating local mucosal immunity in the gut (Malstrom and James, 1998; Klapproth et al., 2000).

The *lifA/efa-1* gene is present in all tested non-O157:H7 EHEC serotype and in related enteropathogens, such as *Citrobacter rodentium* and rabbit EPEC (REPEC) (Klapproth et al., 2000; Nicholls et al., 2000). Although this gene is not physically located in the LEE it has only been observed in *eae*-positive (Nicholls et al., 2000; Vidal et al., 2008) and LEE-positive strains isolated from humans and cattle (Galli et al., 2010). However, the intact efa-1 gene is absent from the *E. coli* O157:H7 strains that have been sequenced (Perna et al., 2001; Janka et al., 2002). *E. coli* O157:H7 possesses a truncated *lifA/efa-1* pseudogene (*efa-1*′) which is located within the pathogenic island O122 and is predicted to encode proteins identical to amino acids 1–433 and 435–710 of open reading frame (ORF) z4332 and the contiguous ORF z4333 respectively (Perna et al., 2001). It has been reported that an *E. coli* O157:H7 mutant carrying a transposon insertion upstream of efa-1′ showed reduced adherence to human colon cells (Stevens et al., 2002), indicating that the truncated Efa-1 protein may have some of the properties of full-length Efa-1, whose last gene (*efa-1*′) could contribute to its virulence (Karmali et al., 2003).

A variety of prevention strategies have been proposed in order to reduce the prevalence of EHEC in animals (Potter et al., 2004; Sargeant et al., 2007; Babiuk et al., 2008; Rozema et al., 2009), and thereby also reduce the incidence of human infections. The use of adjuvants has greatly improved the antigenicity of immunogens. An example is the new generation of immune-stimulation complex (ISCOM) adjuvants such as AbISCO; this is a potent inducer of humoral and cellular immune response against antigens administered via the mucose (Picard et al., 2012). Although mice do not develop the symptoms associated with diarrheal disease observed in human (Roxas et al., 2010), they mice have proved to be useful for EHEC infection and disease (García-Angulo et al., 2014). This includes the C57BL/6 mice (Rhee et al., 2011). In this study, we sought to develop an anti-EHEC DNA-based vaccine utilizing the *efa-1*′ gene, contained in an expression vector, as an innovative strategy to prevent EHEC infections. Similar strategies have been widely used for anti-viral therapy as well as for protection against bacteria and parasites (Dhama et al., 2008). The present study describes the immune response induced by a DNA plasmid encoding sequences of the *efa-1*′ gene in an intranasal C57BL/6 mouse model.

## Materials and methods

### Animals

Eight-week-old female C57BL/6 mice (purchased from Instituto de Salud Pública, Santiago, Chile) were acclimated and randomly assigned to experimental groups. Mice were handled and disposed of according to the guidelines of the Universidad de Concepción Institutional Ethics Committee. According to the experimental procedure, mice were housed in individually cages with free access to food and water in temperature-controlled under 12 h light/12 h dark cycle, an environment free of specific pathogens. The Bioethics and security committee of the Faculty of Biological Sciences in the Universidad de Concepción approved this study. All efforts were made to minimize animal suffering.

### Bacterial strains and culture conditions

The *E. coli* EDL933 (Mohawk and O'Brien, 2011), the prototypical strain *E. coli* O157:H7, was used for experimental infection, for oral inoculation studies, this bacterial strain were amplified in brain heart infusion broth for 18 h at 37°C with shaking. *E. coli* strain DH5α (Life Technology, Gaithersburg, MD) was used to propagate plasmids. *E. coli* DH5α cultures were routinely grown at 37°C in Luria-Bertani broth or agar supplemented, when required, with Kanamycin 100 μg/ml.

### Construction of the DNA vaccine

DNA vaccine constructs expressing efa-1′ from the O-island 122 of *E. coli* O157:H7 were prepared as described below. The coding region for this antigen was PCR amplified from *E. coli* EDL933 chromosomal DNA. Primer sequences are listed in Table S1. PCR products were ligated into the pVAX-cloning vector (Invitrogen). The resulting plasmids were designated pVAX*efa-1*′. pVAX is a plasmid vector designed for use in the development of DNA vaccine and it is characterized by high-level transient expression of the protein of interest in most mammalian cells (Invitrogen, USA). Large amount of endotoxin-free plasmid DNA were prepared and purified using the Endo-free plasmid Giga Kit (Qiagen, Valencia, CA), following the manufacturer's instructions. The analysis of Efa-1′ protein expression was carried out by western blot from Cos7 cell transfected with the plasmid pVAX*efa-1*′. Immunodetection of proteins was carried out by the use of a mouse Flag specific monoclonal antibody (Sigma-Aldrich, Inc.) as the primary antibody (data not shown).

### Purification of EHEC-secreted proteins

Secreted proteins by the Type III Secretion System (TTSS) were prepared from supernatants obtained from *E. coli* EDL933 cultures, as previously described (Niebuhr and Ebel, 2003). Briefly, *E. coli* strain O157:H7 EDL933 was cultivated in Luria-Bertani medium (LB) at 37°C overnight. This culture was then diluted in M-9 minimal medium supplemented with 44 mM NaHCO, 8 mM MgSO_4_, glucose and 0.1% Casamino Acids (Difco Laboratories); these culture conditions optimize the production of Type III Secretion System proteins. The culture was incubated at 37°C in an atmosphere with 5% CO_2_ until the optical density reached 0.7–0.8 at 600 nm. Bacteria were pelleted by centrifugation at 3500 g for 15 min; the supernatant was concentrated by precipitating with trichloroacetic acid (TCA), then 10% (v/v) 100% TCA was added and left overnight at 4°C. We then centrifuged at 4000 g for 20 min, discarded the supernatant, and the pellet was re-suspended in 200 μL Tris-HCl 1.5M. Proteins were stored at −20°C for later use as antigens in ELISA and lymphocyte proliferation assays.

### Immunization

Ten mice per group were anesthetized using a solution of 10 mg/ml ketamine and 250 μg/ml acepromazine and immunized intranasally with a solution containing 50 μg of the recombinant plasmid pVAX*efa-1*′, 12 μg of the adjuvant AbISCO-100® (ISCONOVA AB, Uppsala, Sweden), plus PBS to complete a total volume of 50 μl for the appropriate preparation of the recombinant vector. As negative controls, groups of mice were immunized with empty pVAX vector vaccine, as internal control of plasmid or PBS plus adjuvant respectively. Three doses of vaccine were administered in 14-day intervals (Li et al., 2000). Assays were performed in duplicate; the results are a representative date set.

### Evaluation of antibody response

Mouse serum samples were obtained every 2 weeks prior to each immunization and 2 weeks after the last administration of the DNA vaccine. The presence of serum immunoglobulin (Ig) G, IgM and IgA isotypes with specificity for Efa-1′ was determined by an enzyme-linked immunosorbent assay (ELISA) (Li et al., 2000). For this purpose, 2.5 μg/ml of EHEC-secreted protein diluted in carbonate buffer (pH 9.6) was used to coat the wells of a polystyrene plate at 4°C overnight. The plates were washed three times in PBS, 0.05% Tween 20 (PBST) and the non-specific sites were blocked with 3% gelatin in PBST. After 1 h incubation at 37°C and repeated washing, the plate was incubated with serial dilutions of sera from immunized mice (from 1:500 to 1:20,000) in PBST. They were then added to the ELISA plates and incubated at 37°C for 2 h. Then the plate were repeated washed and anti-mouse isotype HRP-conjugates (1:1000 in PBST, ICN Biomedical, Inc., Ohio, USA) was added, incubated a 37°C for 30 min and washed. After 30 min of incubation, 100 μl of substrate OPD was added (Sigma, USA) to each well. The reaction was stopped with 100 μl of 2 M H_2_SO_4_ and the OD450 was read on a microplate reader (Victor X3. PerkinElmer, USA). The result was expressed as endpoint titer of the last dilution, which gave an optical density at 450 nm of two times above the value of the negative control ± SEM.

In addition, the amount of total specific IgA present in nasal and bronchoalveolar lavages (NAL and BAL respectively) with specificity to EHEC secreted proteins were determined by ELISA (Oñate et al., 2003). Antibody titers were estimated as the reciprocals of the last sample dilution giving an absorbance (A450) value above the cut-off. To compensate for potential variations in the efficiency of recovery of secretory antibodies between animals, the results were normalized according to the total IgA content of the sample. The cut-off value for the assay was calculated as the mean specific OD450 plus standard error of the means (SEM) for 10 NAL or 10 BAL from non-immunized mice assayed at a dilution of 1:10 respectively. Thus, results were expressed as ELISA units (EU), namely the endpoint titer of antigen-specific IgA divided by the total concentration in μg of the IgA present in the sample.

### Splenocyte cultures and lymphocyte proliferation

Two weeks after their last immunization, five mice per group were euthanized. Their spleens were removed and homogenized under aseptic conditions. Single-cell suspensions were prepared according to procedure described (Li et al., 2000). The splenocytes were cultured in RPMI 1640 medium (Sigma), 10% heat-inactivated fetal calf serum (GIBCO BRL), and penicillin-streptomycin (50 UI of penicillin; 50 μg/ml streptomycin) at 37°C with 5% CO_2_ in a 96-well flat-bottom plate at a concentration of 4 × 105 viable cells/well in the presence of no additive (unstimulated control) or one of the following stimulants: (1) 0.2 μg/well of heat-killed *E. coli* strain EDL933 or (2) 1 μg/well of EHEC-secreted proteins. The heat-killed *E.coli* EDL933 wet weight is measured by the cell pellet weight after the centrifugation of a culture obtained as follow; 100 ml of LB inoculated with 1% of ON bacterial culture, was incubated at 37°C with shaking until it reaches an OD_600_ of 0.8 (exponential phase). Then the culture was spun at 20,000 × g by 20 min, suspend to obtain 50 mg/ml (wet cell weight), then the bacteria were killed by heating at 65°C during 1 h, spun at 20,000 × g, and the wet cell pellet was weight and adjusted to the required final concentration.

The cells were cultured for 72 h and pulsed for 8 h with 0.4 μCi of thymidine (50 μCi/mmol; Amersham, UK) per well. The radioactivity incorporated into the DNA of proliferating cells was determined by scintillation counting. Lymphocyte proliferation data were expressed as mean counts per minute of triplicate cultures from a cell pool for each group (five mice per group). In addition, a stimulation index (SI) was calculated for each experimental group by dividing the counts per minute of cells with antigen by the counts per minute of cells without antigen.

### Cytokine mRNA profile

Splenocytes (4 × 105 cells per well) were stimulated with heat-killed *E. coli* strain EDL933 (0.2 μg/well) and EHEC-secreted proteins (1 μg/well) for 9 h (Fu et al., 2013). RNA was isolated from cells using TRIzol® (Invitrogen), as recommended by the manufacturer's instructions, and reverse transcribed to cDNA. Cytokine-specific real-time PCR for interleukin (IL)-4, IL-10 and INF-γ was carried out following standard procedures. The cytokine primer sequences are listed in Table S1. The gapdh gene was used as a control for constitutive gene expression. The amplification reaction was carried out for a total of 40 cycles as follows: 95°C for 30 s, 55°C for 30 s, and 72°C for 30 s, with a pre-cycle of 95°C for 10 min and final extension at 72°C for 5 min. PCR products (5–10 μl) were analyzed by gel electrophoresis. Expression data were normalized against the housekeeping gapdh gene expression profile. Each value was analyzed for statistical difference according to the Bonferroni/Dunn method (Retamal-Díaz et al., 2014).

### Challenge of immunized mice

The protection experiments were performed as described previously (Giulietti et al., 2001). Twenty-four hours before the challenge, mouse were treated with 1 mg/ml streptomycin, which was administered in water which they drank *ad libitum*. Five mice from each group were challenged through oral gavage 6 weeks after the last vaccine dose with 100 μl of a suspension containing 5 × 105 CFU/ml of *E. coli* strain EDL933. Two weeks later, the infected mice were euthanized and their distal and proximal colon were removed under aseptic conditions, homogenized and diluted into plates containing Sorbitol MacConkey agar (Oxoid, Basingstoke, UK), supplemented with 20 μg/ml nalidixic acid (Sigma, St. Louis, USA) and 2.5 μg/ml potassium tellurite (TN-SMAC) in order to determine the number of *E. coli* strain EDL933 CFU per ml. Some non-sorbitol-fermenting colonies were tested by PCR for O157 serogroup (primers are described in Table S1). Bacterial counts of challenged strain were determined in intestinal tissue homogenates and expressed as CFU ml-1 of homogenate. Log10 units of protection were obtained by subtracting the mean log10 CFU for the experimental group from the mean log10 CFU of the corresponding control group.

### Statistical analysis

Data for lymphocyte proliferation, evaluation of the level of antibody, and detection of cytokine were analyzed using ANOVA test with Bonferroni *post-hoc* test (*P*-value of 0.05 or less was considered statistically significant). The data derived from the protection experiment were analyzed using the Two-way ANOVA test and Sidak's multiple comparisons test with a confidence interval of 95% (α = 0.05).

## Results

### Immune response of mice vaccinated with DNA vaccines

**Figure 1:**
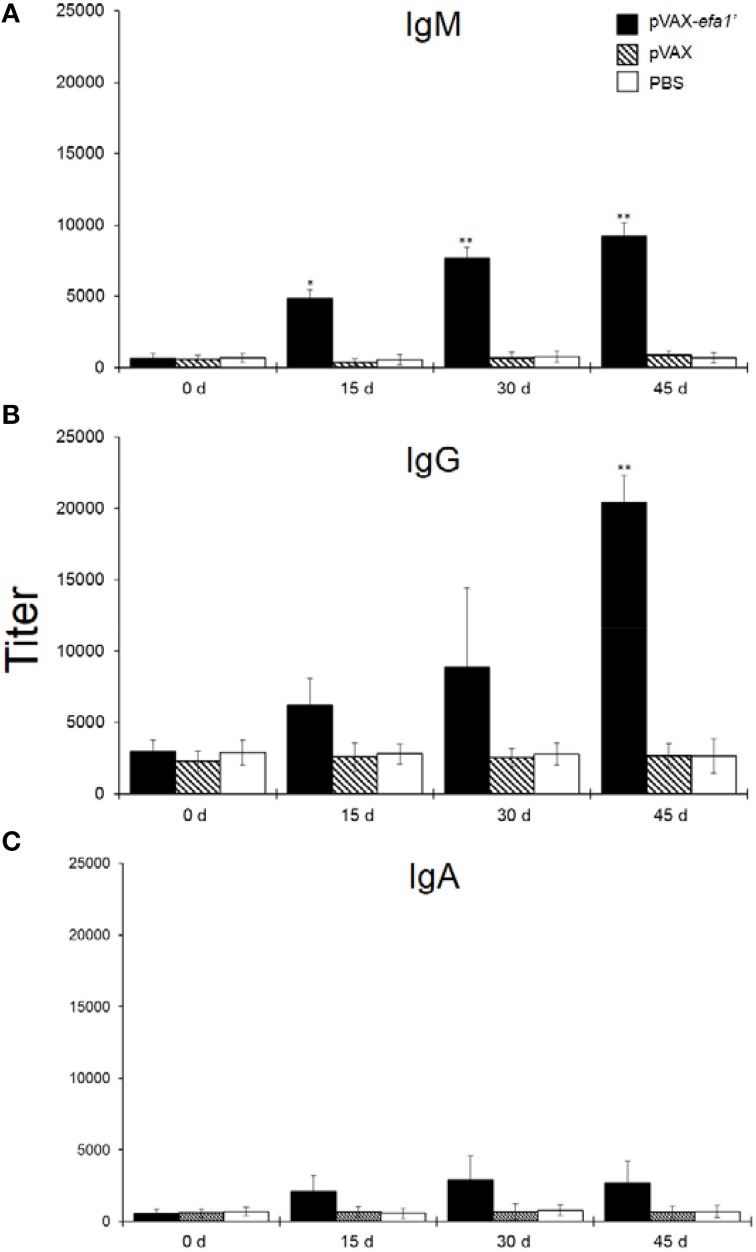
**Efa-1′-specific serum antibodies**. Five mice in each group were immunized by i.n. route with pVAXefa-1′, control pVAX and PBS. Serum samples were obtained on days 0, 15, 30, and 45 post-immunization, and Efa-1′-specific IgM **(A)**, IgG **(B)**, and IgA **(C)** levels in the samples were quantified. Endpoint titers are expressed as retrograde values of the last dilution that gave an OD_450_ of two times above the value of the negative control ± SEM. These results are representative of data from two independent experiments. Statistical significances are represented by asterisks (^*^P < 0.05, and ^**^P < 0.01, as compared to the control PBS group).

We examined the immune response induced in mice intranasally immunized with pVAX*efa-1*′ (co-administered with adjuvant AbISCO). Systemic anti-Efa-1′ IgM, IgG and IgA were detected. As shown in Figure 1A, 15 days after priming the sera from mice immunized with pVAX*efa-1*′ contained a significant titer of anti-Efa-1′ IgM (*P* < 0.05 in comparison to the negative control groups PBS or pVAX). On days 30 and 45 after priming, the titers of anti-Efa-1′ IgM were again significantly higher as compared to the negative control groups PBS or pVAX (*P* < 0.01). Later, Efa-1′ IgG was determined in sera of vaccinated animals. The result showed that 15 and 30 days after immunization with pVAX*efa-1*′ the titer of anti-Efa-1′ IgG was higher, but not significantly, in comparison with the values obtained in mice from the negative control groups (Figure 1B; *P* > 0.05). At day 45, the level of IgG showed a higher increase compared with other days. This was statistically significant compared to the negative control groups (*P* < 0.01). The titer of anti-Efa-1′ IgA present in sera from mice immunized with the vector pVAX*efa-1*′ exhibited slight variation throughout the experiment, with no significant difference when compared with values from mice in the negative control groups (Figure 1C).

**Figure 2:**
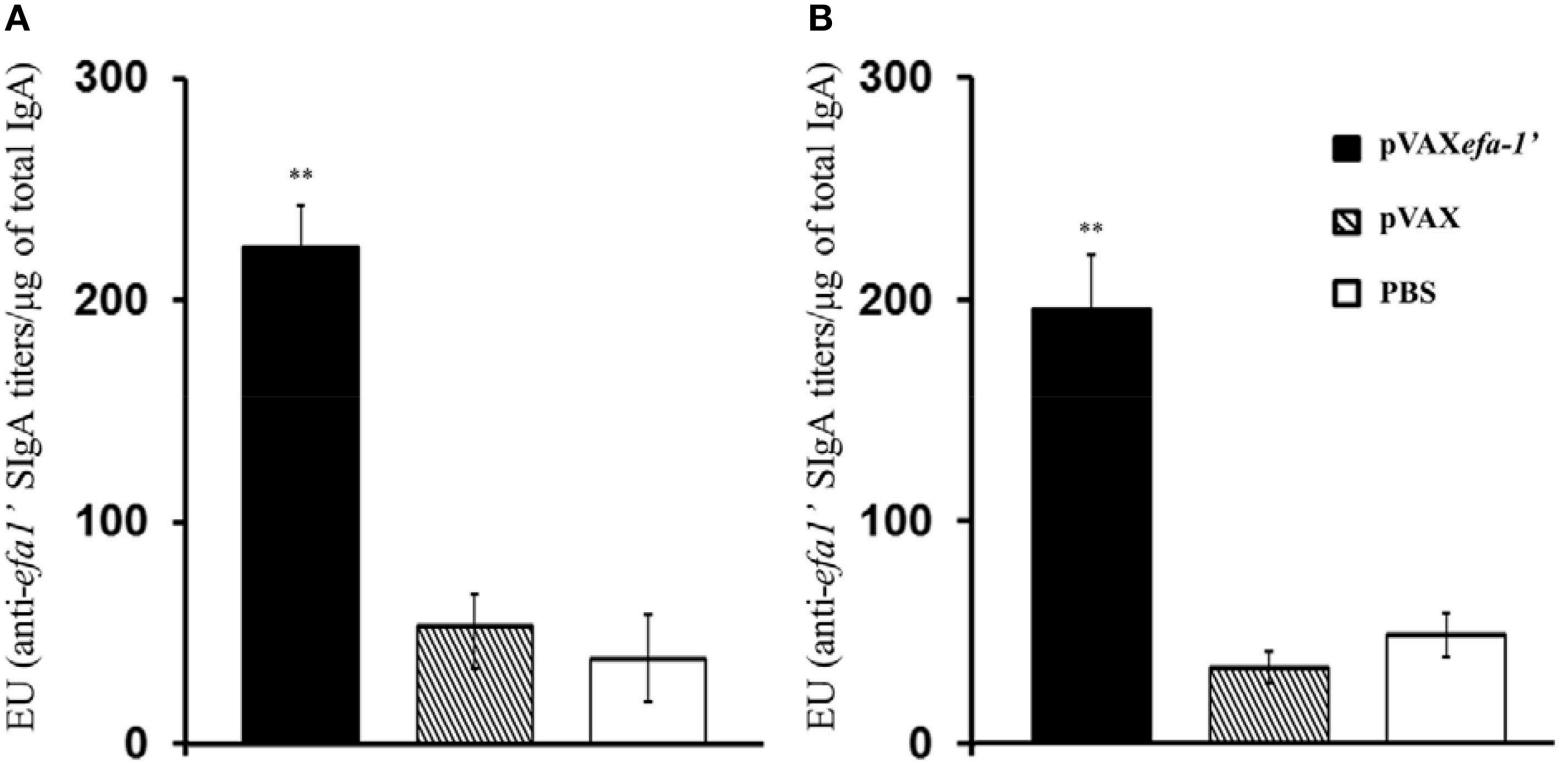
**Efa-1′-specific mucosal sIgA antibodies in nasal lavages (A) and bronchoalveolar lavage (B) fluids**. Efa-1′-specific antibody titers were estimated as the reciprocals of the last sample dilution giving an A450 value above the cut-off. Results were normalized according to the total IgA content of the sample. Results are expressed as ELISA units (EU), namely, the endpoint titer of SOD-specific IgA divided by the total concentration in μg of IgA present in the sample. Data are shown as mean ± SEM of two experiments. The statistical significances are represented by ^**^P < 0.01.

In order to evaluate the induction of mucosal responses in vaccinated animals, EHEC-secreted specific sIgA titres were determined directly from nasal lavage (NAL) and bronchoalveolar lavages (BAL) (Figure 2). Results indicated that vaccination with pVAX*efa-1*′ induced significantly higher levels of antigen-specific mucosal IgA production in both NAL (Figure 2A) and BAL (Figure 2B) as compared to control mice receiving PBS or pVAX (*P* < 0.01).

**Figure 3:**
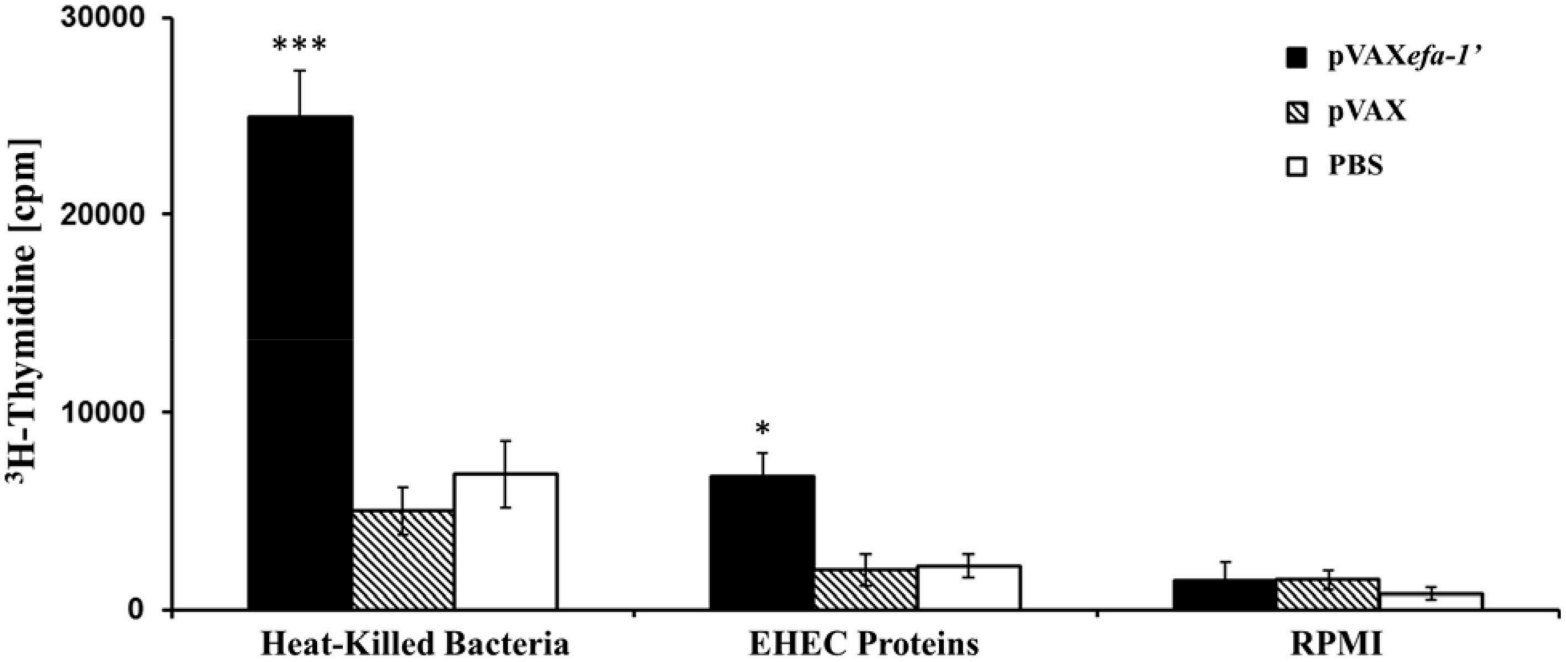
**Lymphocyte proliferation assay**. C57BL/6 mice were immunized with pVAX*efa-1*′, pVAX, or PBS. Efa-1′-T-cell proliferative responses were measured 2 weeks after the last immunization by [^3^H] thymidine incorporation. Splenocyte derived from animals in each group were pooled, and 4 × 10^5^ cells for wells were restimulated *in vitro* with heat-killed *E. coli* (0.2 μg/well) or EHEC-secreted protein (1 μg/well). Each bar indicates the average number of counts per minute for triplicate cultures of cells ± standard deviation (error bar) obtained from five mice per group. Statistical significances are represented by asterisks (^*^P < 0.05, and ^***^P < 0.001).

In most cases the generation of an efficient and long lasting immune response requires the induction of a T-helper cell sub-set. Thus, we opted for examining the cellular mediated immunity (CMI) response to heat-killed *E. coli* and EHEC-secreted protein in the vaccinated mice. For this purpose, we measured the proliferative response of splenocyte following *in vitro* restimulation with the corresponding antigen (Figure 3). Splenocytes from mice immunized with pVAX*efa-1*′ exhibited a significant proliferative response to heat-killed *E. coli* EDL933 (*P* < 0.01 in comparison to the PBS or pVAX groups), with a stimulation index of 5.23. Equivalently, when the splenocytes from mice immunized with pVAX*efa-1*′ were stimulated *in vitro* with EHEC-secreted proteins, they showed a statistically significant increase compared to control groups (*P* < 0.05), with a stimulation index of 4.34. T cells from mice of all groups had similarly high levels of proliferative response to the mitogen Concanavalin A (data not shown).

**Figure 4:**
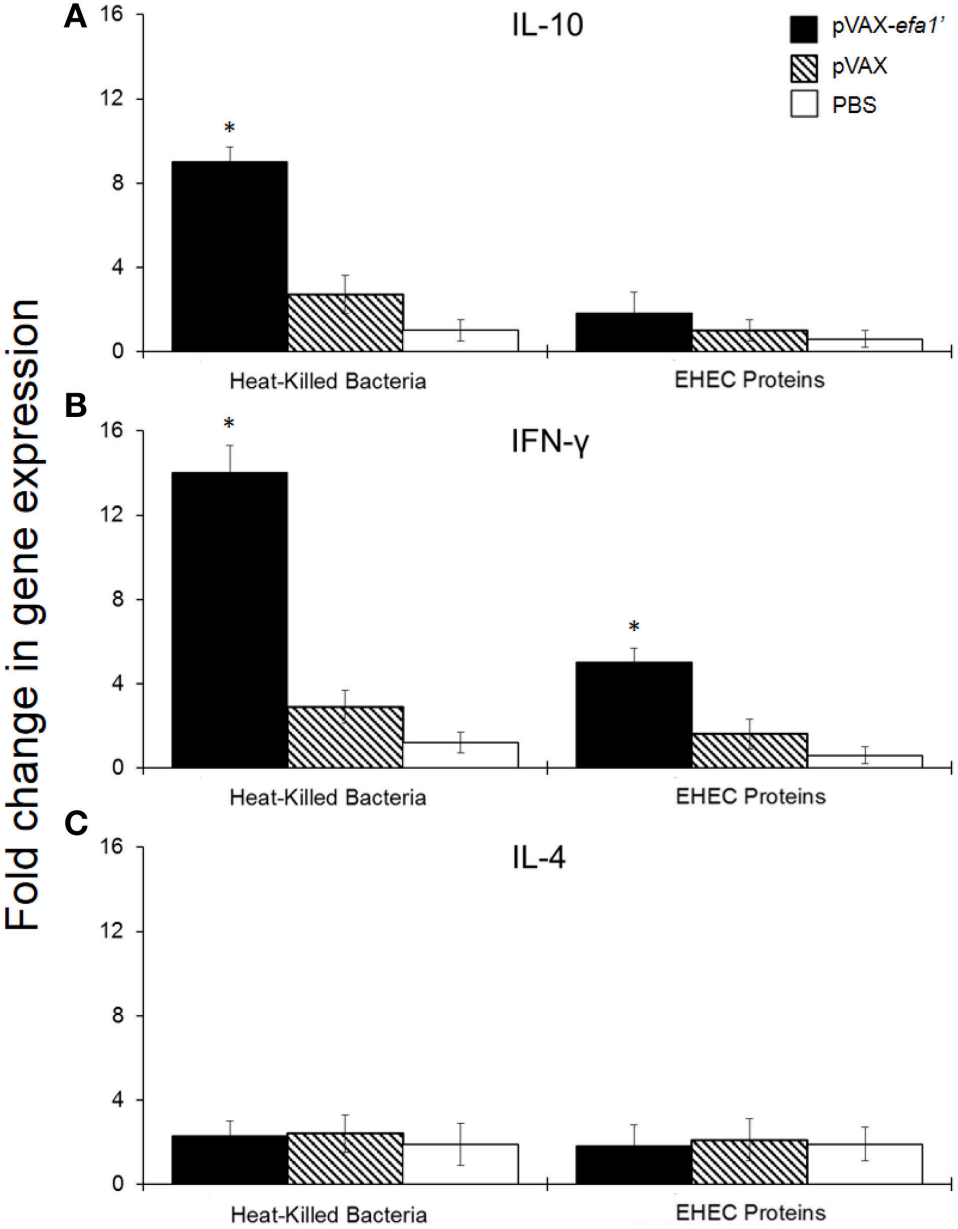
**Relative mRNA expression of IL-10 (A) and IFN-γ (B) and IL-4 (C) produced by mouse splenocytes**. Fold variation of encoded cytokines is shown 9 h after stimulation with 0.2 μg/well of heat-killed *E. coli* and 1 μg/well of EHEC secreted proteins. ^*^Significant differences with respect to the group inoculated with PBS (*P* < 0.05).

To analyse the stimulated immune response in more detail, we also evaluated the cytokine production by spleen-derived lymphocytes after restimulation with the antigen. We used reverse transcription-polymerase chain reaction (RT-PCR) to quantify IL-10, IFN-γ and IL-4 mRNA levels in spleen-cell culture from pVAX*efa-1*′-inmunized animals. By RT-PCR we observed an 8-fold increase in IL-10 mRNA expression (Figure 4A) and a 15-fold increase in IFN-γ mRNA expression (Figure 4B), at 9 h post-stimulation with heat-killed *E. coli*, in splenocytes from pVAX*efa-1*′-vaccinated mice. This result was statistically significant compared to expression levels in unstimulated cells (*P* < 0.05). Upon stimulation with EHEC-secreted proteins, splenocytes from pVAX*efa-1*′-vaccinated mice expressed IL-10 and IFN-γ, but only IFN-γ levels were significant compared to unstimulated cells (*P* < 0.05) (Figure 4B). No significant IL-4 mRNA was detected in any of the splenocyte-stimulated groups (Figure 4C).

### Efficacy of pVAX*efa-1*′ immunization in generating protective immunity against *E. coli* EDL933

Immune protection experiments were carried out by challenging vaccinated and control mice by oral gavage with virulent *E. coli* strain EDL933. The level of infection was evaluated by bacterial counts in intestinal tissue homogenates and expressed as CFU/ml-1 of homogenate. The results showed that immunization with pVAX*efa-1*′ induced a high degree of protection, 3.51 log10 less bacterial in this group, compared with the PBS negative control groups, (*P* < 0.001) (Table 1).

**Table 1:** **Protection of mice against challenge with *E. coli* O157:H7 after immunization with DNA vaccine**.

**Vaccine**	**Log_10_ CFU of *E. coli* O157:H7 strain EDL933 in intestine (mean ± SD)**	**Log_10_ units of protection**
Saline control	4.29 ± 1.01	0.00
pVAX	3.81 ± 0.43	0.48
pVAX*efa-1*′	0.78 ± 0.52^**^	3.51

Five mice for each group were challenged orally with 10^4^ CFU of E. coli O157:H7 strain EDL933 2 week prior to euthanasia. Bacterial counts of challenged strain were determined in intestinal tissue homogenates, five mice per group, and expressed as CFU ml^−1^ of homogenates.

** P < 0.001 compared to the negative controls. Statistical differences were determined using the Two-way ANOVA test and Sidak's post-hoc test with a confidence interval of 95% (α = 0.05).

## Discussion

EHEC were first recognized as a cause of human disease in 1982 (Asahara et al., 2004) and are associated with diarrhea, hemorrhagic colitis, and life-threatening haemolytic uremic syndrome (Riley et al., 1983). The incidence of EHEC varies by age group, with the highest incidence occurring in children under 15 years of age (0.7 cases per 100,000 in USA). Potential complications associated with EHEC include HUS, the principal cause of acute kidney failure in children. Most cases of HUS are caused by *E. coli* O157:H7 (Tarr et al., 2005). In the case of EHEC infection, antibiotic therapy is not usually recommended because antibiotics may induce toxin release from the pathogen as a result of an increased systemic exposure to the adverse effects of the potent nephrotoxin (Wong et al., 2000; Zhang et al., 2000) and there is a need for new strategies to control EHEC colonization, including from animal reservoirs. Particular focus should be placed on protecting cattle, the largest reservoir worldwide, as well as swine, another significant reservoir in many countries (Fremaux et al., 2006; Kagambèga et al., 2012).

The proteins encoded within the LEE have been used as subunit vaccines to generate an increase in serum antibody production and lymphocyte proliferation in murine (Momtaz et al., 2013) and cattle models (Cataldi et al., 2008). Not all strains of EHEC and EPEC harbor the same virulence factors. For example, STEC strains lacking intimin (eae), isolated from patients with hemorrhagic colitis or HUS, may carry *iha, efa-1, lpfA*, and/or *saa* genes (Vidal et al., 2008). Therefore, it is necessary to find other potential immunogens capable of inducing or improving a wide-range of protective immunity to EHEC. Based on our studies Efa-1′ meet the required criteria, because it is conserved among various EHEC serogrups and elicit a strong immune response. Intramuscular immunization of cattle with the truncated recombinant protein (Efa-1′) has been shown to induce a humoral response to the specific antigen, but not protective immunity (McNeilly et al., 2010). Nevertheless, it cannot be excluded that under different conditions, such as route of administration or dose of challenge, different results could be obtained.

The use of immunostimulating complexes (ISCOMs) has been shown to induce robust cellular and humoral immune responses against antigens administered intranasally (van Diemen et al., 2007). Prominent IL-12 production by innate immune cells is a characteristic reaction induced by ISCOMs, promoting the development of a strong Th1 response. After intranasal or intestinal mucosal administration, the ISCOM induces a strong specific mucosal IgA response on local and remote mucosal surfaces, with stimulation of the secretion of proinflammatory cytokines such as IL-1 and TNF-α, or anti-inflammatory cytokines such as IL-10 (Hu et al., 2001).

DNA vaccination has already proved affective against *E. coli* O157:H7 (Morein et al., 2004). We examined the effectiveness of the DNA vaccine transport efa-1′ administered together with AbISCO adjuvant, in generating immunity and protection against experimental infection with the *E. coli* strain EDL933 in mice. With EHEC-secreted proteins as antigen, the plasmid pVAX*efa-1*′ induced the highest titers of IgM and IgG production compared with negative control groups pVAX and PBS. A strong antibody response against LEE-encoded proteins has been reported after experimental infection with EHEC O157:H7 (Morein et al., 2004). Nevertheless, it is known that in natural EHEC infections in cattle, the serum antibody response is not essential to generate an efficient protective response (Shariati Mehr et al., 2012). At the mucosal level, intranasal immunization with our vaccine resulted in the production of higher antigen-specific IgA titers (sIgA) in BAL than in NAL. Several studies have demonstrated that such locally produced antibodies, mainly sIgA, which prevents the binding of bacteria and toxin action on epithelial cells, most likely provide protective immunity (Kaper et al., 2004). These results are correlated with the high rate of lymphocyte stimulation observed in the splenocytes of animals immunized with the *efa-1*′ gene, with the production of INF-γ, IL-10 and significant protection. Contrarily, it has been reported that various strains of EHEC are capable of inhibiting the INF-γ pathway through inhibition of Stat-1 phosphorylation in epithelial cells, contributing to immune evasion by these microorganisms (Bretschneider et al., 2007). The high expression of INF-γ in vaccinated mice may offset the inhibitory effects caused by EHEC infection. Moreover, the expression of the anti-inflammatory cytokine IL-10 may contribute to the significant mucosal immune response observed by immunization with pVAX*efa-1*′. B cells are the major source of IgA precursor cells. They undergo a class switch recombination to IgA secreting cells, which are heavily dependent on cytokines secreted by activated T cells, such as IL-10, which in lamina propria promotes conversion of sIgA B cells to mature sIgA secreting plasma cells (Ho et al., 2012), potentiating a Th2-biased immunological response. Moreover, high level of IL-10 expression has been reported in mice vaccinated with Shiga toxins (Stxs) fusion proteins from EHEC (Fagarasan et al., 2010), demonstrating that production of IL-10 is a natural response against *E. coli* pathogenic infection. Thus, immunization with pVAX*efa-1*′ induces significant production of IFN-γ associated with immune protection against microbial pathogens, with the production of IL-10 cytokine that is crucial in preventing inflammation, and thus protecting tissues from damage. This is essential for the maintenance of gut homeostasis and the recovery of the intestinal epithelial barrier. Additionally, this protects gut mucosal tissues from colitis (Cai et al., 2011). Therefore, the joint action of sIgA in mucosa together with the high production of INF-γ and IL-10 observed in pVAX*efa-1*′ immunized mice, may explain the high protective response observed in this group. Further, the production of IL-10 in conjunction with IFN-γ could be interpreted as a regulatory immune mechanism that prevents uncontrolled TH type 1 immune responses that could be potentially harmful (Li et al., 2014) o beneficial agianst to EHEC (Ghasemi et al., 2014).

## Conclusions

This study shows that genetic vaccines containing *efa-1*′ from *E. coli* O157:H7 result in the induction of mucosal and systemic immune responses. This approach is able to confer efficient protection against challenge with the enterohemorrhagic *E. coli* EDL933 in the mouse model studied. Overall, these results indicate that mucosal inoculation with DNA vaccines is a valid vaccination approach for the induction of immuno-mediated protection against EHEC infections.

## Author contributions

RR, writing and discussion of the results, perform cytokines qPCR assays. AR, writing the paper, performs immunization trials, antibodies evaluation by ELISA and lymphocyte proliferation assays. DS, he was performing the recombinant protein, cloning and immunization. PF, conducted evaluation tests of immunity at mucosal level and statistical analysis of the results. FD, cloning, discussion, and writing of the manuscript. JS, discuss and determine the growth culture conditions for optimal protein purification from *E. coli* EDL933. GO, he analyzed the efa-1′ gene sequence in enterohemorrhagic *E. coli* isolates from patients, animal and foods. RV, define the truncated region of enterohemorrhagic *E. coli* (EHEC) factor for adherence-1 gene (efa-1′) for protective immunogenic analysis, programming and monitoring the experiments, collecting the results, performing the analysis, and discussion and writing the manuscript. AO, programming and monitoring the experiments, collecting the results, performing the analysis and discussion and writing the manuscript. RV and AO are principal investigators at the FONDECYT grant that funded this work.

### Conflict of interest statement

The authors declare that the research was conducted in the absence of any commercial or financial relationships that could be construed as a potential conflict of interest.
