# Supplementary material for: Vaccination with DNA Encoding Truncated Enterohemorrhagic Escherichia coli (EHEC) Factor for Adherence-1 Gene (efa-1′) Confers Protective Immunity to Mice Infected with E. coli O157:H7
Source: Front Cell Infect Microbiol. 2016 Jan 20;5:104. doi: 10.3389/fcimb.2015.00104 (PMC4718977; doi:10.3389/fcimb.2015.00104)
Supplement: Supplementary file 1 [file Table1.DOCX]

**Supplementary Information**

**Table S1**. **Primers used in this study**

| **Gene** | **Use** | **Sequence^a^** | **Restriction enzime** | **Reference** |  |
| --- | --- | --- | --- | --- | --- |
| *efa-1’* | Cloning F  R | AGGATCCATTATGGGACTGCCAGAGAAAGTTCTTT  ACTCGAGCTAAAAAAATGAATTAAACATGT | *BamHI*  *XhoI* | This study |  |
| *il-4* | Detection F  R | ACAGGAGAAGGGACGCCAT  ATCGAAAAGCCCGAAAGAGT |  | (29) |  |
| *il-10* | Detection F  R | GGTTGCCAAGCCTTATCGGA  CGAGGTTTTCCAAGGAGTTG |  | (29) |  |
| *ifn-γ* | Detection F  R | TCAAGTGGCATAGATGTGGAAGAA  TGGCTCTGCAGGATTTTCATG |  | (29) |  |
| *gapdh*  *O157rfb* | Detection F  R  Detection F  R | ACCCAGAAGACTGTGGATGG  CCCCAGCATCAAAGGTAGAA  AAGATTGCGCTGAAGCCTTTG  CATTGGCATCGTGTGGACAG |  | (29)  (58) |  |

^a^ Restriction endonuclease cleavage sites are underlined.
